# Supplementary material for: Comparative transcriptome profiling provides insights into the growth promotion activity of Pseudomonas fluorescens strain SLU99 in tomato and potato plants
Source: Front Plant Sci. 2023 Jul 18;14:1141692. doi: 10.3389/fpls.2023.1141692 (PMC10393259; doi:10.3389/fpls.2023.1141692)
Supplement: Supplementary file 5 [file DataSheet_1.pdf]

Supplementary Material

Comparative Transcriptome Profiling Provides Insights into the Growth Promotion Activity of *Pseudomonas fluorescens* strain SLU99 in Tomato and Potato Plants

Nurul Atilia Shafienaz binti Hanifah[1,2], Farideh Ghadamgahi[1], Samrat Ghosh[1], Rodomiro Ortiz, [1] Stephen C. Whisson,[3] Ramesh R. Vetukuri[1*], and Pruthvi B. Kalyandurg[1*]

*** Correspondence:**

Pruthvi B. Kalyandurg

pruthvi.balachandra@slu.se

Ramesh R. Vetukuri

ramesh.vetukuri@slu.se

Supplementary Table 1. Analysis of soil nutrients and characteristics after amendment with selected rhizobacteria and growth of tomato and potato plants. TN: total nitrogen, P: phosphorus, K: potassium, EC: electrical conductivity, SOM: soil organic matter.

Treatment	TN (mg/kg)		Available P (mg/l)		Available K (mg/l)		pH		EC (mS/cm)		SOM (%)	
	Tomato	Potato	Tomato	Potato	Tomato	Potato	Tomato	Potato	Tomato	Potato	Tomato	Potato
***P. fluorescens* SLU99**	7080	5780	36	29	140	15	5.9	6.5	1.7	1.4	42.7	43
***S. rubidaea* EV23**	6190	6000	34	27	140	11	5.9	6.4	2.1	1.3	43.4	48
***S. rubidaea* AV10**	6360	6030	34	27	130	14	5.9	6.4	2.4	1.4	39.6	44
***S. plymuthica* S412**	5510	6190	34	28	150	11	5.8	6.5	2.3	1.3	41.0	44
**Control**	6850	6160	32	28	130	9	5.9	6.5	2.3	1.2	42.9	46

Supplementary Table 2. Overview of differentially expressed transcription factor (TF) families identified in tomato and potato plants upon treatment with culture filtrate of *Pseudomonas fluorescens* SLU99.

TF family	Differentially Expressed TFs			
	Tomato Root	Potato Root	Tomato leaf	Potato leaf
AP2	0	5	0	0
ARR-B	1	0	2	2
B3	4	4	1	3
bHLH	11	23	19	8
bZIP	1	2	1	0
CO-like	0	6	1	3
DBB	0	5	5	0
ERF	16	18	15	4
GATA	2	6	5	3
GRAS	3	6	3	1
HD-ZIP	11	23	1	6
HSF	4	1	5	2
LBD	6	13	6	6
MIKC_MADS	14	8	1	2
MYB	21	16	16	8
MYB_related	3	8	2	5
NAC	2	12	3	8
NF-Y	4	4	3	1
TALE	2	4	1	1
TCP	0	5	0	2
Trihelix	1	1	1	1
WOX	1	1	0	0
WRKY	4	8	1	4
ZF-HD	1	3	0	0

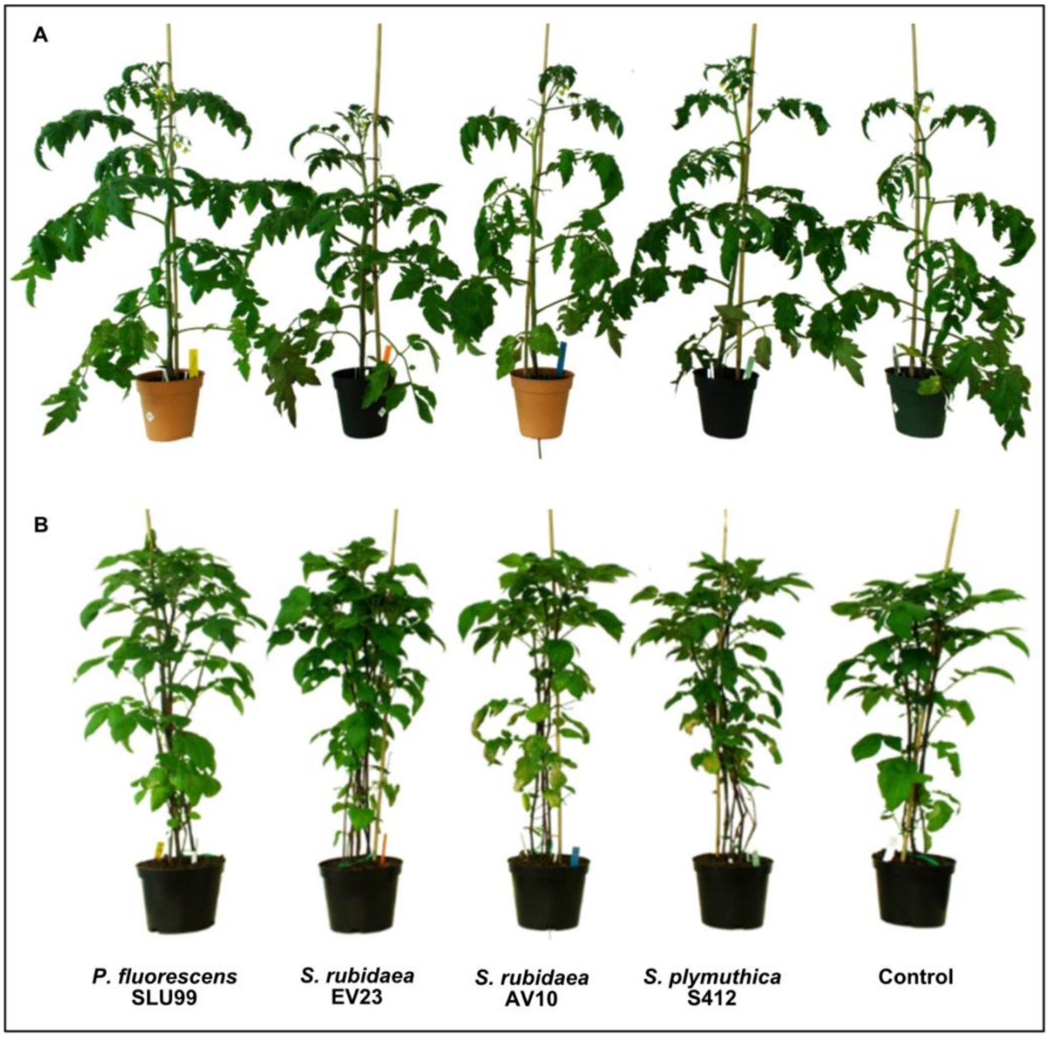

Supplementary Figure 1. Plant height after treatment with different bacteria. (A) Tomato plants 42 days after transplanting (B) Potato plants 53 days after planting.

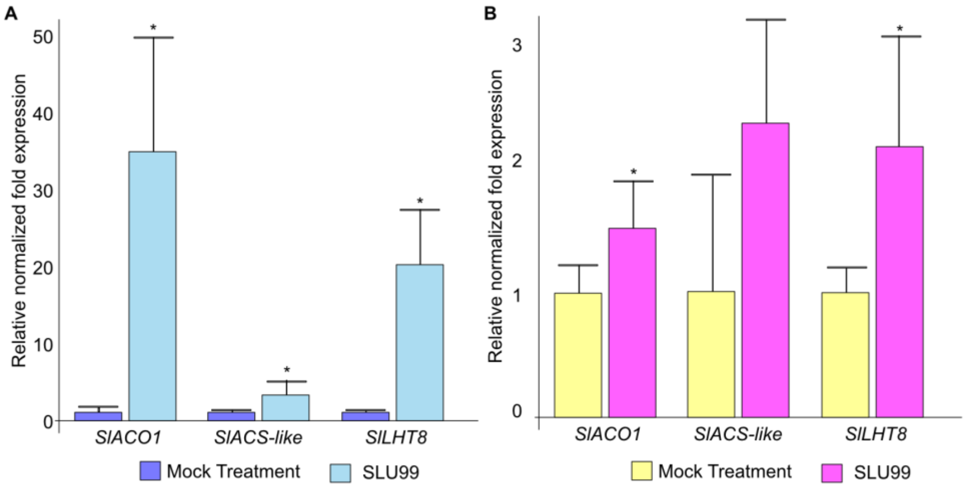

Supplementary Figure 2. Relative expression of SlACO1, SlACS and SlLHT8 in roots (A) and leaves (B) of tomato plants treated with mock or SLU99 supernatant.
